# Evaluation of Herbal Methionine and *Mangifera Indica* Against Lead-induced Organ Toxicity in Broilers

**DOI:** 10.4103/0971-6580.75864

**Published:** 2011

**Authors:** D. Udaya Lakshmi, K. Adilaxmamma, A. Gopala Reddy, V. Vykunta Rao

**Affiliations:** Department of Pharmacology and Toxicology, College of Veterinary Science, Rajendranagar, Hyderabad - 30, Andhra Pradesh, India; 1 Department of Pharmacology and Toxicology, College of Veterinary Science, Tirupati-517 502, Andhra Pradesh, India; 2 Department of Pharmacology and Toxicology, Teaching Veterinary Clinical Complex, Tirupati - 517 502, Andhra Pradesh, India

**Keywords:** Hepatotoxicity, herbal methionine, lead, *Mangifera Indica*, oxidative stress

## Abstract

Lead toxicity was studied in male broiler chicks (*Cobb* strain) of a day-old age. The chicks were randomly divided into six groups consisting of 15 in each group. Group 1 was maintained as basal diet control and group 2 was kept on lead at 300 ppm in feed throughout 5 wk as toxic control without any treatment. Groups 3 and 4 were maintained on herbal methionine at 1.4 g/kg feed + *Mangifera Indica* at 0.1% in feed, respectively. Groups 5 and 6 were treated with lead + herbal methionine and lead + *M. indica*, respectively, for the 5 wk. The concentration of thiobarbituric acid reactive substances (TBARS) and protein carbonyls, and the activities of superoxide dismutase (SOD) and catalase in liver and kidney revealed a significant (*P*<0.05) increase, while there was a significant (*P*<0.05) decrease in the concentration of reduced glutathione (GSH) in liver and kidney, and hepatocytic membrane ATPases and cytochrome P_450_ (CYP_450_) in liver in the lead toxic control group. Treatment with herbal remedies in groups 5 and 6 resulted in a marked improvement in all the above parameters as compared to those of lead toxic control. Thus, it is concluded that lead induces biological damage by means of oxidative stress and the herbs in test offered better amelioration. The beneficial effects of herbal methionine and *Ma. indica* may be attributed to their antioxidant, anti-stress and hepatoprotective principles.

## INTRODUCTION

Lead is one of the widely dispersed toxic substances, and sources of lead in environment include lead paints, automobiles, industrial lead emission and lead in food/feed and water. However, lead gasoline combustion in vehicles has accounted for as much as 90% of the total anthropogenic sources of environmental lead.[1] Such accumulated lead is toxic in most of its chemical forms, whether it is inhaled or ingested through water or feed. Lead affects the metabolism of other minerals and has affinity for bone, where it acts by replacing calcium. Thus, the highest concentrations of lead are usually found in bone, kidney and liver.[2] Studies have reported that lead has a potential for inducing oxidative stress and acts as a catalyst in the oxidative reactions of biological molecules by producing free radicals or reactive oxygen species (ROS; hydroxyl radicals, peroxyl radicals, alkyl radicals, etc.). Lead interferes with different antioxidant defenses like glutathione peroxidase (GSH-Px), glutathione reductase (GSH-R), catalase, superoxide dismutase (SOD) and reduced glutathione (GSH). Reduced amounts of antioxidants may contribute to progression of liver damage and damage to other organ systems including kidney and nervous system.[3] Several herbs have been reported to counter the peroxidative stress in biological system due to several stressors including pesticides, mycotoxins and heavy metals. Keeping the above facts in view, an experimental study was planned to evaluate herbal methionine and *Mangifera Indica* against experimental lead toxicosis in broilers.

## MATERIALS AND METHODS

A total of 90 sexed male broiler chicks (*Cobb* strain) of day-old age were randomly divided into six groups consisting of 15 in each group. The chicks were provided with feed and water ad *libitum* throughout the experiment. The groups were maintained as per the following feeding schedule for 5 wk in order to study the antioxidant role of herbal methionine (combination of *Cicer arienticum, Triticum sativum, Phaseolus mungo, Mucuna puriens* and *Allium cepa*) and *M. indica* against lead-induced oxidative damage.

Group 1: Basal diet control
Group 2: Lead toxic control (300 mg/kg feed)
Group 3: Herbal methionine control (25% above normal level in feed, i.e., 1.4 g/kg feed)
Group 4: *M. indica* control (0.1% in feed)
Group 5: Lead + herbal methionine
Group 6: Lead + **M. indica**

The birds were sacrificed at the end of 5^th^ week and tissues were collected for the assay of reduced glutathione (GSH),[4] superoxide dismutase (SOD),[5] catalase,[6] protein carbonyls[7] and thiobarbituric acid reactive substances (TBARS)[8] in liver and kidney homogenates. The activities of Na^+^/K^+^ ATPase, Mg^2+^ATPase[9] and cytochrome P_450_ (CYP_450_)[10] were estimated in the liver homogenate. The data were analyzed by one-way analysis of variance (ANOVA) using statistical package for social sciences (SPSS) version 10. *P*<0.05 was considered as significant.

## RESULTS AND DISCUSSION

The concentration of TBARS [μM of malondialdehyde (MDA)/mg protein] in liver and kidney of basal diet control (group 1) was 2.37±0.13 and 1.90±0.12, respectively, which was significantly (*P*<0.05) increased in lead toxic control group 2 at the end of 5^th^ week. Groups 5 and 6 that were supplemented with herbs revealed significantly (*P*<0.05) decreased concentration of TBARS as compared to group 2. The concentration of protein carbonyls (nM/100 mg protein) in liver and kidney of basal diet control (group 1) was 18.79±1.02 and 10.13±0.75, respectively, which was significantly (*P*<0.05) increased in lead toxic control group 2 at the end of 5^th^ week. Groups 5 and 6 that were supplemented with herbs revealed significantly (*P*<0.05) decreased concentration of protein carbonyls as compared to lead control. The concentration of GSH (μM/mg protein) in liver and kidney of basal diet control (group 1) was 9.29±0.62 and 14.88±1.58, respectively, which was significantly (*P*<0.05) decreased in lead toxic control group 2 at the end of 5^th^ week. Groups 5 and 6 that were supplemented with herbs revealed significantly (*P*<0.05) increased concentration of GSH as compared to lead control. All these parameters in groups 3 and 4 were comparable to that of basal diet control [Tables 1 and 2].

**Table 1:** Results of oxidative stress and enzymes in liver

Group	TBARS (*μ*M of MDA/mg protein)	GSH (μM/mg protein)	SOD (U/mg protein)	Catalase (*μ*g of H_2_O_2_ decomposed/ mg protein/min)	Protein carbonyls (nM/100 mg protein)	Na^+^/K^+^ ATPase (μM of Pi released/ mg protein/30 min)	Mg^2+^ ATPase (*μ*M of Pi released/mg protein/30 min)	CYP_450_ (*μ*M/ mg microsomal protein)
1	2.37±0.13^a^	9.29±0.62^c^	13.36±0.57^d^	11.65±0.58^cd^	18.79±1.02^a^	6.81±0.50^c^	13.53±0.59^c^	0.09±0.01^c^
2	5.35±0.18^c^	2.41±0.37^a^	5.22±1.63^a^	4.48±1.45^a^	37.93±1.60^c^	2.73±0.19^a^	10.16±0.44^a^	0.03±0.01^a^
3	2.39±0.14^a^	8.04±1.11^c^	11.66±0.92^cd^	11.62±0.67^c^	19.36±0.82^a^	6.75±0.38^c^	13.35±0.42^c^	0.09±0.01^c^
4	1.89±0.08^a^	9.89±0.53^c^	14.15±1.08^d^	13.23±1.82^d^	18.07±1.83^a^	6.99±0.76^c^	14.73±0.83^c^	0.10±0.02^c^
5	3.99±0.38^b^	5.37±0.79^b^	8.58±0.72^b^	8.10±0.42^b^	30.14±0.97^b^	4.28±0.50^b^	11.80±0.28^b^	0.06±0.01^b^
6	3.89±0.32^b^	5.95±0.70^b^	10.13±0.53^bc^	9.19±0.80^bc^	26.57±3.44^b^	4.79±0.39^b^	11.81±0.22^b^	0.06±0.01^b^

Values are Mean±SE (*n* = 8); one-way ANOVA (SPSS). Means with different alphabets as superscripts differ significantly (*P*<0.05); TBARS, thiobarbituric acid reactive substances; MDA, malondialdehyde; GSH, glutathione; SOD, superoxide dismutase

**Table 2:** Results of oxidative stress in kidney

Group	TBARS concentration (*μ*M of MDA/mg protein)	GSH concentration (*μ*M/mg protein)	SOD activity (U/mg protein)	Catalase activity (*μ*g of H_2_O_2_ decomposed/mg protein/min)	Protein carbonyls concentration (nM/100 mg protein)
1	1.90±0.12^a^	14.88±1.58^c^	9.02±0.47^c^	10.50±0.34^cd^	10.13±0.75^a^
2	5.33±0.91^c^	3.11±0.57^a^	1.59±0.13^a^	4.07±0.26^a^	34.22±1.20^c^
3	2.02±0.13^a^	12.45±0.32^bc^	11.02±0.94^d^	9.61±0.20^cd^	13.14±0.93^a^
4	1.17±0.14^a^	15.48±1.58^c^	9.95±0.31^cd^	11.97±1.76^d^	9.76±1.22^a^
5	3.76±0.74^b^	9.99±1.18^b^	6.17±0.51^b^	7.19±0.24^b^	20.59±1.54^b^
6	3.58±0.57^b^	10.11±1.37^b^	7.04±0.82^b^	7.35±0.24^b^	19.53±4.35^b^

Values are Mean±SE (*n* = 8); one-way ANOVA (SPSS). Means with different alphabets as superscripts differ significantly (*P*<0.05); TBARS, thiobarbituric acid reactive substances; MDA, malondialdehyde; GSH, glutathione; SOD, superoxide dismutase

The activity of SOD (U/mg protein) in liver and kidney in basal diet control (group 1) was 13.36±0.57 and 9.02±0.47, respectively, which was significantly (*P*<0.05) decreased in lead toxic control group 2 at the end of 5^th^ week. Groups 5 and 6 that were supplemented with herbs revealed significantly (*P*<0.05) increased activity of SOD as compared to that of lead control. The activity of catalase (μg of H_2_O_2_ decomposed/mg protein/min) in liver and kidney of basal diet control (group 1) was 11.65±0.58 and 10.50±0.34, respectively, which was significantly (*P*<0.05) decreased in lead toxic control group 2 at the end of 5^th^ week. Groups 5 and 6 that were supplemented with herbs revealed significantly (*P*<0.05) increased activity of catalase as compared to lead control [Tables 1 and 2]

The concentration of TBARS and protein carbonyls in liver and kidney was significantly increased in toxic control group, while the activities of SOD and catalase, and the concentration of GSH in liver and kidney were reduced, suggesting the ongoing peroxidative stress and compromised antioxidant defense mechanisms. Enhanced oxidative stress contributes to lead-induced toxicity, where restoration of a cells’ antioxidant capacity appears to provide a partial remedy. Several studies are underway to determine the effect of antioxidant supplementation following lead exposure. Data suggest that antioxidants may play an important role in abating some hazards of lead.[2] It has been reported earlier that lead impairs the antioxidant defenses of the cells and renders them more susceptible to oxidative attacks.[11,12] All the changes in the antioxidant defense profile were significantly reversed when treated with herbal methionine and *Ma. indica*. The beneficial effects of herbal methionine are attributed to antioxidant and anti-stress principles, namely, *Mu. pruriens*[13] and *A. cepa,*[14] besides other constituent principles. *Ma. indica* has proven antioxidant potential.[15] The aqueous stem bark extract of *Ma. indica* L. has been reported to have antioxidant properties. In a study, Hernandez *et al*,[16] reported that *Ma. indica* extract attenuated accumulation of reactive oxygen species (ROS) and intracellular free Ca^2+^, and consequently, down-regulated CD95L mRNA expression and CD95-mediated activation-induced cell death (AICD). *Mu. pruriens* has the ability to scavenge DPPH (α,α-diphenyl-β-picrylhydrazyl) radicals, ABTS radicals and reactive oxygen species. It significantly inhibits the lipid oxidation[17] and effectively scavenges hydroxyl radicals (OH’) and superoxide anion radicals (O_2_’^−^).[18] Treatment with *Mu. pruriens* decreased the levels of lipid peroxidation and increased the levels of glutathione (GSH), superoxide dismutase (SOD) and catalase (CAT).[13]

The activity of Na^+^/K^+^ ATPase and Mg^2+^ ATPase (μM of Pi released/mg protein/30 min) in liver of basal diet control group 1 was 6.81±0.50 and 13.53±0.59, respectively, which was significantly (*P*<0.05) decreased in lead toxic control group 2 at the end of 5^th^ week. Groups 5 and 6 that were supplemented with herbs revealed significantly (*P*<0.05) increased activity of ATPases as compared to lead control. The activity in groups 3 and 4 was comparable to that of basal diet control. The activity of CYP_450_ (μM/mg microsomal protein) in liver of basal diet control group 1 was 0.09±0.01, which was significantly (*P*<0.05) decreased in lead toxic control group 2 at the end of 5^th^ week. Groups 5 and 6 that were supplemented with herbs revealed significantly (*P*<0.05) increased activity of CYP_450_ as compared to lead control. The activity in groups 3 and 4 was comparable to that of basal diet control [Table 1].

The activities of Na^+^/K^+^ ATPase, Mg^2+^ ATPase and CYP_450_ were significantly reduced in the liver of lead toxic control. Lipid peroxidation is a natural and deleterious process. The decrease in the levels of Na^+^/K^+^ ATPase, Mg^2+^ ATPase and CYP_450_ could be due to enhanced lipid peroxidation by free radicals in lead treated groups. Since these membrane-bound enzymes are sulfhydryl (SH) group containing and are lipid-dependent, any alteration in the membrane lipids owing to lipid peroxidation may significantly alter their activity. The treated groups revealed slight improvement in the membrane bound enzymes though the activities were not exactly similar to those of controls. It has been reported that dietary lead alters fatty acid composition and membrane peroxidation in chick liver microsomes.[19] These findings could be further substantiated from the results of the present study, which revealed a significant increase in lipid peroxidation (indicated by increased TBARS) in liver of the lead toxic control as compared to the remaining groups.

The results of the present study enunciated that supplementation of herbal methionine and *Ma. indica* could significantly reverse the oxidative stress induced by lead. The beneficial actions of herbal methionine and *Ma. indica* may be attributed to their antioxidant, anti-stress and hepatoprotective principles.

## References

[1] Flora SJ, Dube SN (1995). Chelating agents for the treatment of lead intoxication. Indian J Environ Toxicol.

[2] Gurer H, Ercal N (2000). Can antioxidants be beneficial in the treatment of lead poisoning?. Free Radic Biol Med.

[3] Farrukh A, Iqbal A, Zafar M (2006). Antioxidant and free radical scavenging properties of twelve traditionally used Indian medicinal plants. Turkish J Biol.

[4] Moron MS, Depierre JW, Mannervik B (1979). Levels of glutathione, glutathione reductase and glutathione S transferase in rat lung and liver. Biochim Biophys Acta.

[5] Madesh M, Balasubramanian KA (1998). Microtiter plate assay for superoxide dismutase using MTT reduction by superoxide. Indian J Biochem Biophys.

[6] Asru KS (1972). Colorimetric assay of catalase. Anal Biochem.

[7] Levine RL, Garland D, Oliver CN, Amici A, Climent I, Lenz AG (1990). Determination of carbonyl content in oxidatively modified proteins. Methods Enzymol.

[8] Subramanian KA, Manohar M, Mathan VI (1988). An unidentified inhibitor of lipid peroxidation in intestinal mucosa. Biochim Biophys Acta.

[9] Kiku N, Shiusuke K, Makoto N (1967). Adenosine triphosphatase activity of erythrocyte membrane in hereditary spheocytosis. Life Sci.

[10] Choi SJ, Kim M, Kim SI, Jeon JK (2003). Microplate assay measurement of cytochrome P_450_ - carbon monoxide complexes. J Biochem Mol Biol.

[11] Ercal N, Gurer OH, Aykin-Burns N (2001). Toxic metals and oxidative stress part I: Mechanisms involved in metal-induced oxidative damage. Curr Top Med Chem.

[12] Hultberg B, Anderson A, Isaksson A (2001). Interaction of metals and thiols in cell damage and glutathione distribution; Potentiation of mercury toxicity by dithiothreitol. Toxicol.

[13] Rajeshwar Y, Kumar GP, Gupta M, Maunder UK (2005). Studies on in vitro antioxidant activities of methanol extract of *Mucuna pruriens (Fabaceae)* seeds. Eur Bull Drug Res.

[14] Prakash D, Singh BN, Upadhyay G (2007). Antioxidant and free radical scavenging activities of phenols from onion (*Allium cepa*). Food Chem.

[15] Stoilova I, Gargova S, Stoyanova A, Ho L (2005). Antimicrobial and antioxidant activity of the polyphenol mangiferin. Herba Polonica.

[16] Hernandez P, Delgado R, Walczak H (2006). *Mangifera Indica* L.extract protects T cells from activation-induced cell death. Int Immunopharmacol.

[17] Dhanasekaran M, Tharakan B, Manyam BV (2008). Anti Parkinson drug-*Mucuna pruriens* shows antioxidant and metal chelating activity. Phytother Res.

[18] Siddhuraju P, Becker K (2003). Studies on antioxidant activities of mucuna seed (*Mucuna pruriens* var *utilis*) extract and various non-protein amino/imino acids through *in vitro* models. J Sci Food Agril.

[19] Knowles SO, Donaldson WE (1996). Dietary lead alters fatty acid composition and membrane peroxidation in chick liver microsomes. Poult Sci.

